# Association of circulating exosomal miR-122 levels with BAT activity in healthy humans

**DOI:** 10.1038/s41598-019-49754-1

**Published:** 2019-09-13

**Authors:** Yuko Okamatsu-Ogura, Mami Matsushita, Jussiaea Valente Bariuan, Kazuki Nagaya, Ayumi Tsubota, Masayuki Saito

**Affiliations:** 1 Laboratory of Biochemistry, Faculty of Veterinary Medicine, Hokkaido University, Sapporo, 060-0818 Japan; 2 Department of Nutrition, School of Nursing and Nutrition, Tenshi College, Sapporo, 065-0013 Japan

**Keywords:** Metabolic syndrome, Obesity

## Abstract

Brown adipose tissue (BAT) plays an important role in body fat accumulation and the regulation of energy expenditure. Since the role of miRNAs in the pathogenesis of obesity and related metabolic diseases is contentious, we analyzed exosomal miRNAs in serum of healthy subjects with special references to BAT activity and body fat level. Forty male volunteers aged 20–30 years were recruited. Their BAT activity was assessed by fluorodeoxyglucose positron emission tomography and computed tomography after 2 h of cold exposure and expressed as a maximal standardized uptake value (SUVmax). Exosomal miRNA levels was analyzed using microarray and real-time PCR analyses. The miR-122-5p level in the high BAT activity group (SUV ≧ 3) was 53% lower than in the low BAT activity group (SUVmax <3). Pearson’s correlation analysis revealed that the serum miR-122-5p level correlated negatively with BAT activity and the serum HDL-cholesterol, and it correlated positively with age, BMI, body fat mass, and total cholesterol and triglyceride serum levels. Multivariate regression analysis revealed that BAT activity was associated with the serum miR-122-5p level independently of the other parameters. These results reveal the serum exosomal miR-122-5p level is negatively associated with BAT activity independently of obesity.

## Introduction

Brown adipose tissue (BAT) is a tissue specialized for non-shivering thermogenesis and thereby plays a role in the regulation of energy expenditure and body fat level not only in small rodents and also in humans^1–3^. The recruitment of BAT by chronic cold exposure or intake of some thermogenic compounds results in the reduction of adiposity both in rodents and humans^4,5^. Thus, BAT has attracted much attention as a target for the treatment of obesity and obesity-related metabolic disorders.

MicroRNAs (miRNAs) are endogenous non-coding RNAs of about 22 nucleotides in length and function to inhibit gene expression by targeting mRNAs for translational repression or cleavage^6^. MiRNA acts not only in the intracellular space, but also in distant recipient cells since it can be transported by exosome, a small vesicle that can deliver molecules through circulation. It is recognized that miRNA is involved in the pathogenesis of obesity and related metabolic disorders, including diabetes mellitus^7–9^. In fact, the circulating levels of several exosomal miRNAs are positively or negatively correlated with obesity and insulin resistance in humans^10–12^.

It is also proposed that miRNA plays a role in brown adipogenesis and thermogenic energy expenditure. For example, miR-133 has reported as a negative regulator of brown adipocyte differentiation^13^. Genetic ablation of miR-133 resulted in the enhancement of brown adipogenesis and the improvement of insulin sensitivity and glucose tolerance^14^. On the other hand, miR-196a has been reported as a positive regulator of brown adipogenesis, and its overexpression in adipose tissue led to resistance to obesity due to an increase in BAT activity and energy expenditure^15^. Furthermore, the studies using adipocyte-specific knockout of Dicer or Dgcr8, regulators of miRNA processing, revealed the crucial role of miRNAs produced in adipose tissue in the maintenance and differentiation of brown adipocytes^16,17^. Besides these intracellular miRNAs expressed in brown adipocytes, it was reported that blood miR-99b secreted from BAT modulates gene expression and metabolism in the liver^18^. Despite the increasing evidence for the regulatory roles of miRNA in BAT functions and obesity, most of them come from the studies in mice, and the relationship among miRNA, BAT, and body fatness is poorly understood in humans. Thus, an idea that the reported association of circulating miRNA with obesity in humans^10–12^ may be mediated through BAT is possible. In the present study, we analyzed exosomal miRNAs in serum samples obtained from healthy human subjects, particularly with respect to the BAT activity and body fatness.

## Results

The fluorodeoxyglucose positron emission tomography and computed tomography (FDG-PET/CT) revealed a large variation in the BAT activity among the 40 subjects with SUVmax ranging from 0.5 to 21.0. The subjects were then divided into low and high BAT activity groups based the median SUVmax value of 3.0. As shown in Table 1, age, body fat, and blood parameters were not significantly different between the two groups.

**Table 1:** Subject profiles.

	All subjects (n = 40)	High-BAT (n = 20)	Low-BAT (n = 20)	*p-value* High vs. Low
Age, years	22.3 ± 2.3	21.9 ± 1.9	22.8 ± 2.7	0.26
Weight, kg	61.5 ± 8.7	59.6 ± 6.7	63.4 ± 10.1	0.18
BAT, SUV	6.2 ± 6.5	11.0 ± 6.0	1.4 ± 0.8	<0.001***
BMI, kg m^−2^	21.0 ± 2.7	20.5 ± 1.8	21.5 ± 3.4	0.27
Body fat mass, kg	9.9 ± 4.0	9.0 ± 3.0	10.8 ± 4.7	0.17
Visceral fat area, cm^2^	35.5 ± 17.6	33.6 ± 17.1	37.5 ± 18.2	0.49
Subcutaneous fat area, cm^2^	86.4 ± 68.9	74.4 ± 51.4	98.4 ± 82.3	0.27
Total cholesterol, mg dl^−1^	178.3 ± 33.0	176.9 ± 35.6	179.7 ± 31.1	0.79
HDL-cholesterol, mg dl^−1^	62.4 ± 13.6	65.0 ± 15.1	59.8 ± 11.8	0.23
Triglycerides, mg dl^−1^	75.7 ± 46.4	67.5 ± 35.8	84.0 ± 54.8	0.26
Glucose, mg dl^−1^	79.7 ± 5.1	81.0 ± 6.1	78.5 ± 3.5	0.12
Insulin, m UI^−1^	4.9 ± 3.0	4.9 ± 2.5	5.0 ± 3.5	0.89
HOMA-IR	0.98 ± 0.63	0.99 ± 0.56	0.98 ± 0.71	0.96

Mean ± SD.

Abbreviations: BAT, brown adipose tissue; SUV, standardized uptake value; BMI, body mass index.

***P < 0.001.

**Figure 1:**
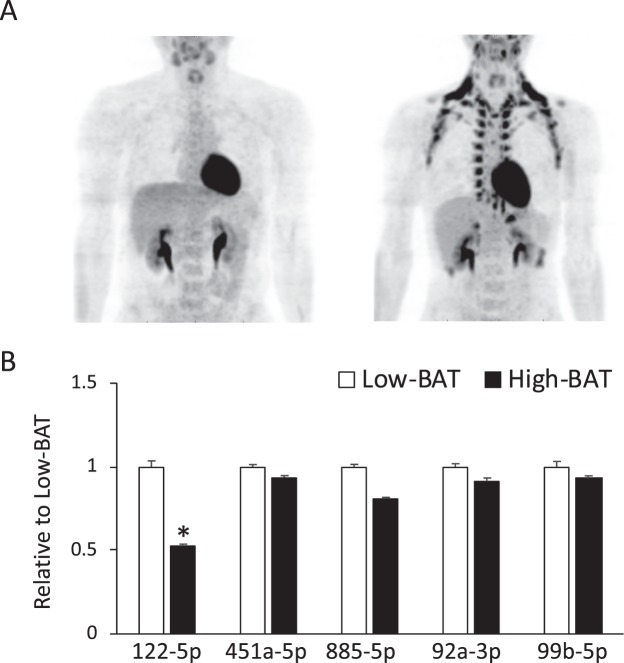
Serum exosomal miRNA levels in subjects with a high-BAT and low-BAT. (**A**) Representative FDG-PET/CT images of subjects with low (left) and high (right) BAT activities, respectively. (**B**) Exosomal miRNA levels quantified by qPCR. Subjects were divided into two groups with low (SUV < 3.0) and high (SUV ≧ 3) BAT activities. MiRNA expression was normalized to the level of Cel-miR-39-3p, which was added to the exosome samples as a spiked internal control before miRNA extraction and expressed as relative to the low BAT activity group. Values are shown as the mean ± SE and n = 20 per group. Significance was assessed using a Welch’s two sample t-test. *P < 0.05 compared to the low BAT activity group.

To identify the circulating miRNAs that may be associated with the BAT activity, we first performed a microarray analysis using serum samples from 2 representative individuals each in the low- and high- BAT groups. When the criteria were set to identify circulating levels that differed by more than 1.5-fold between the groups, only a limited number of miRNAs were selected as candidates. Among these, we picked up three miRNAs, miR-122-5p, miR-451a-5p, and miR-885-5p, and tried confirming the microarray results by a real-time PCR analysis of the serum samples obtained from all 40 subjects. Other candidates are shown in Supplementary Fig. S1. The miR-122-5p level in the high BAT activity group was 53% of that in the low activity group (Fig. 1), while there was no significant difference in the circulating levels of miR-451a-5p and miR-885-5p between the two groups. We also measured the circulating levels of miR-92a-3p, which have been reported to be associated with BAT activity in humans^19^, and of miR-99b-5p, which has been reported to be secreted from BAT in mice^18^; however, there was no significant difference in these two miRNAs between the groups.

Pearson’s correlation analyses revealed that the serum miR-122-5p level correlated negatively with the BAT activity (R = −0.360, P = 0.022; Table 2) and HDL-cholesterol but positively with BMI, body fat mass, the visceral and subcutaneous fat area, and the total serum cholesterol and triglycerides. There were also significant correlations of miR-885-5p levels with BMI and body fat mass; however, neither miR-451a-5p nor miR-885-5p levels exhibited significant correlation with other parameters, including BAT activity. Univariate and multivariate logistic regression analyses revealed that BAT activity was associated with serum miR-122-5p levels independently of the body fatness (Table 3).

**Table 2:** Pearson Correlation for miRNAs and individual profile.

	miR-122-5p	miR-451a-5p	miR-885-5p	miR-92a-3p	miR-99b-5p
BAT, SUV	−0.360*	−0.063	−0.075	−0.148	−0.220
	(0.022)	(0.702)	(0.649)	(0.363)	(0.174)
BMI, kg m^−2^	0.536***	0.209	0.318*	0.335*	0.339*
	(<0.001)	(0.198)	(0.045)	(0.034)	(0.032)
Body fat mass, kg	0.528***	0.09	0.315*	0.401*	0.252
	(<0.001)	(0.581)	(0.047)	(0.010)	(0.117)
Visceral fat Area, cm^2^	0.441***	0.144	0.29	0.347*	0.266
	(<0.001)	(0.378)	(0.069)	(0.027)	(0.098)
Subcutaneous fat area, cm^2^	0.567^***^	0.027	0.261	0.338^*^	0.263
	(<0.001)	(0.872)	(0.104)	(0.033)	(0.102)
Total cholesterol, mg dl^−1^	0.440**	0.084	0.271	0.332*	0.015*
	(0.004)	(0.609)	(0.091)	(0.036)	(0.034)
HDL-cholesterol, mg dl^−1^	−0.323*	−0.242	−0.027	−0.389*	−0.327*
	(0.041)	(0.134)	(0.870)	(0.013)	(0.039)
Triglycerides, mg dl^−1^	0.575***	0.007	0.294	0.368*	0.217
	(<0.001)	(0.968)	(0.066)	(0.019)	(0.180)
Glucose, mg dl^−1^	0.135	0.078	−0.113	0.102	−0.048
	(0.410)	(0.636)	(0.490)	(0.532)	(0.769)
Insulin, mUI^−1^	0.299	0.314	−0.001	0.302	0.134
	(0.061)	(0.048)	(0.995)	(0.058)	(0.414)
HOMA-IR	0.273	0.308	−0.024	0.301	0.112
	(0.089)	(0.053)	(0.884)	(0.059)	(0.490)

Correlation coefficient (p value).

Abbreviations: BAT, brown adipose tissue; SUV, standardized uptake value; BMI, body mass index.

*P < 0.05, **P < 0.01, ***P < 0.001.

**Table 3:** Univariate and multiple regression analysis for miR-122-5p.

	Univariate regression		Multivariate regression		
	R	P	β	Standardized β	P
**miR-122-5p**					
BAT, SUV	−0.36	0.022*	−0.024	−0.276	0.045*
BMI, kg m^−2^	0.536	<0.001***	0.009	0.041	0.555
Body fat mass, kg	0.437	0.005**	0.002	0.02	0.727
Visceral fat area, cm^2^	0.441	0.004**	−0.004	−0.118	0.815
Subcutaneous fat area, cm^2^	0.567	0.006**	0.001	0.14	0.506
Total cholesterol, mg dl^−1^	0.44	0.005**	0.002	0.143	0.352
HDL-cholesterol, mg dl^−1^	−0.323	0.042*	0.006	−0.151	0.523
Triglycerides, mg dl^−1^	0.575	<0.001***	0.003	0.243	0.160

Abbreviations: BAT, brown adipose tissue; SUV, standardized uptake value; BMI, body mass index.

*P < 0.05, **P < 0.01, ***P < 0.001.

## Discussion

We found that the BAT activity is negatively associated with serum miR-122-5p levels. This miRNA is known to be liver-specific and thereby a useful biomarker for various liver diseases^20^. In addition, its circulating level has been reported to correlate with obesity. For example, Ortega *et al*. investigated the pattern of circulating miRNAs in obesity and found that the miR-122 level was significantly elevated in individuals who are obese and decreased after a surgery-induced weight loss^10^. Subsequently, it was reported that the miR-122 level is also associated with insulin resistance^11,12^, a non-alcoholic fatty liver^21^, a future risk for metabolic syndrome and type-2 diabetes^22^. Consistent with these observations, our study revealed significant positive correlations between the miR-122 level and BMI and adiposity. Moreover, the miR-122 level was found to correlate negatively with the BAT activity. These results seem consistent with the well-established negative correlation between the BAT activity and body fatness^1–3,23^. However, the multivariate regression analysis revealed that the BAT activity, but not body fatness, is an independent determinant for miR-122. Thus, the previously reported correlation between the miR-122 level and obesity may be largely due to the BAT activity rather than body fatness itself.

Previously, it was reported that a circulating miR-92a-3p level significantly correlates with the BAT activity in humans^19^. In our study, miR-92a-3p levels did not differ between the low and the high BAT activity groups and exhibited no correlation with the BAT activity. One of the reasons for this discrepancy might be due to differences in the subject profiles; body fatness differed considerably among the subjects in the previous report, whereas we recruited subjects with comparable body fat masses so that any possible impact of body fatness would be minimized. Sex differences may also be involved, since both male and female subjects were included in the previous study, whereas all subjects in this study were male. Further study will be needed to clarify the reasons for these differences.

Several miRNAs have been reported to be involved in the regulation of brown adipogenesis inside of brown adipocytes of mice^13–17^. On the other hand, miR-99b has been reported as a circulating exosomal miRNA secreted from BAT^18^, but we found no significant correlation between the miR-99b level and BAT activity. This may be due to differences in BAT adipocytes of humans and mice; that is, the mouse BAT consists of classical brown adipocytes, whereas the BAT of human adults seems to consist of both classical brown and inducible beige adipocytes or mainly of beige adipocytes^24,25^.

The question of how BAT activity is associated with the circulating level of miRNA specifically expressed in the liver is unclear. The possibility exists that some BAT-derived factors^26^ may act on the liver to regulate the expression or the secretion of miR-122-5p into exosomes. Conversely, the circulating miR-122-5p level may affect metabolism of other tissues. In fact, suppression of miR-122-5p by intravenous injection of an antisense miRNA in mice resulted in a decrease in the total serum cholesterol level and an increase in hepatic fatty acid oxidation^27^. Circulating miR-122-5p has been suggested to regulate the metabolism of glucose and branched chain amino acids by lowering the expression of enzymes such as aldolase A, pyruvate kinase, and branched chain keto-acid dehydrogenase kinase^28,29^. Thus, low concentrations of circulating miR-122-5p in subjects with a high BAT activity may partly explain their low blood cholesterol and glucose levels^30,31^.

In conclusion, the serum exosomal miR-122-5p level is negatively associated with the BAT activity, independently of obesity in humans. Further study is needed to identify the mechanism and the pathophysiological relevance for this correlation, but our results suggest that exosomal miR-122-5p could be a blood marker for the evaluation of human BAT activity.

## Materials and Methods

### Subjects

All subjects gave their informed consent for inclusion before they participated in the study. The study was conducted in accordance with the Declaration of Helsinki and the trial was registered with the UMIN Clinical Trials Registry (UMIN000031255). The subjects recruited for this study were 40 healthy male volunteers aged 20–30 years. They were carefully instructed regarding the purpose of the study and gave their informed consent to participate. The protocol was approved by the institutional review boards of Tenshi College (No. 2015-25) on September 18, 2015.

### FDG-PET/CT

All subjects received 18FDG-PET/CT after 2 h of mild cold exposure as described previously^23^. Briefly, after fasting for 6–12 h, subjects entered an air-conditioned room at 19 °C wearing light clothing (usually a T-shirt and underwear) and intermittently placed their feet on a cloth-wrapped ice block (usually for 4 min every 5 min). After 1 h, they were given an intravenous injection of 18F-FDG (1.66–5.18 MBq per kg body weight) and were kept under the same cold conditions for an another hour. Whole-body PET/CT scans were performed using a PET/CT system (Aquiduo, Toshiba Medical Systems, Odawara, Japan) in a room maintained at 24 °C. PET and CT images were co-registered and analyzed using a VOX-BASE workstation (J-MAC System, Sapporo, Japan). BAT activity in the neck region was quantified by calculating the maximal standardized uptake value (SUVmax), defined as the radioactivity per milliliter within the region of interest divided by the injection dose in megabecqurels per gram of body weight.

### Anthropometric and body fat measurements and blood analysis

The body fat mass was estimated by multifrequency bioelectric impedance method (Full Body Sensor Body Composition Monitor and Scale HBF-361; Omron, Kyoto, Japan). The visceral and subcutaneous fat areas at the L4–L5 abdominal level were estimated from the CT images.

Blood samples were taken before the cold exposure and analyzed by laboratory testing service (SRL, Tokyo, Japan), or used for exosome isolation.

### Extraction of circulating miRNA

Exosomes were isolated from the serum using a Total Exosome Isolation kit (Thermo Fisher Scientific, Gaithersburg, MD) according to the manufacturer’s instructions. Briefly, 50 µl of the serum was mixed with 10 µl of isolation reagent and incubated for 30 min at 4 °C. After centrifugation at 10,000 *g* for 10 min at 4 °C, the resulting pellet was dissolved in 100 µl of PBS, and miRNA was extracted using a miRNA easy kit (Qiagen, Hilden, Germany). Briefly, 500 µl of QIAzol reagent was added to 100 µl of isolated exosomes and incubated for 5 min at room temperature. After the addition of 5 µl of 5 nM Cel-miR-39-3p spike-in (Qiagen) and 100 µl of chloroform, samples were centrifuged at 12,000 *g* for 15 min at 4 °C. The upper aqueous phase was transferred to a fresh tube, and 1.5 volumes of ethanol were added. The sample was then applied to columns and washed. MiRNA was eluted in 30 µl of nuclease-free water. The quantity of miRNA was measured using Qubit (Thermo Fisher Scientific) and microRNA assay kit (Thermo Fisher Scientific).

### Microarray analysis

Exosomal miRNAs from 4 subjects, each from the low- and the high-BAT groups, were subjected to comprehensive analysis of miRNA expression patterns using microarray-based technology. Microarray analysis was performed by Hokkaido System Science Co., Ltd (Sapporo, Japan) using SurePrint G3 Human 8 × 60 K microarrays (Agilent Technologies, Palo Alto, CA). The scan was performed using an Agilent Technologies Microarray Scanner, and each spot was digitized using an Agilent Feature Extraction version 12.0.3.1 software. To identify the miRNAs that were differentially expressed between the two groups, data were imported into the GeneSpring GX software (Agilent Technologies) for analysis.

### Analysis of miRNA by real-time PCR

The extracted total miRNA was reverse transcribed using a Mir-X miRNA First Strand Synthesis kit (Clontech, Palo Alto, CA). Briefly, 3 µl of miRNA was mixed with a mRQ enzyme and incubated at 37 °C for 1 h and then at 85 °C for 5 min. After the addition of 100 µl of deionized water, 2 µl of the sample was used for real-time PCR using a fluorescence thermal cycler (Light Cycler system, Roche Diagnostics, Mannheim, Germany), FastStart Essential DNA Green Master (Roche Diagnostics) probe, and primers specific to each miRNA. Data were normalized to Cel-miR-39-3p levels.

### Data analysis

Statistical analyses were performed using R version 3.5.1. Differences between the low and the high BAT groups were analyzed by Welch’s two sample *t*- tests. Simple correlations were assessed by using the Pearson correlation. Univariate and multivariate logistic regression analyses were used to test the significance of the association between the BAT activity and other factors. P-values < 0.05 were considered to be statistically significant.

## Supplementary information

Supplementary Figure S1
