# Supplementary material for: Association of circulating exosomal miR-122 levels with BAT activity in healthy humans
Source: Sci Rep. 2019 Sep 13;9:13243. doi: 10.1038/s41598-019-49754-1 (PMC6744505; doi:10.1038/s41598-019-49754-1)

Association of circulating exosomal miR-122 levels with BAT activity in healthy humans

Yuko Okamatsu-Ogura[1*], Mami Matsushita[2], Jussiaea Valente Bariuan[1], Kazuki Nagaya[1], Ayumi Tsubota[1] and Masayuki Saito[1]

[1] *Laboratory of Biochemistry, Faculty of Veterinary Medicine, Hokkaido University, Sapporo 060-0818, Japan*

[2] *Department of Nutrition, School of Nursing and Nutrition, Tenshi College, Sapporo 065-0013, Japan*

Supplementary Figure S1

List of miRNAs which showed more then 1.5-fold difference between the low and high BAT groups

Low BAT > High BAT

mirR name	miRBase Accession No
hsa-miR-6090	MIMAT0023715
hsa-miR-6125	MIMAT0024598
hsa-miR-3960	MIMAT0019337
hsa-miR-6087	MIMAT0023712
hsa-miR-6821-5p	MIMAT0027542
hsa-miR-2861	MIMAT0013802
hsa-miR-1246	MIMAT0005898
hsa-miR-630	MIMAT0003299
hsa-miR-7150	MIMAT0028211
hsa-miR-638	MIMAT0003308
hsa-miR-5703	MIMAT0022496
hsa-miR-6869-5p	MIMAT0027638
hsa-miR-3656	MIMAT0018076
hsa-miR-1915-3p	MIMAT0007892
hsa-miR-320c	MIMAT0005793
hsa-miR-7704	MIMAT0030019
hsa-miR-5787	MIMAT0023252
hsa-miR-4466	MIMAT0018993
hsa-miR-3679-5p	MIMAT0018104
hsa-miR-6891-5p	MIMAT0027682
hsa-miR-7110-5p	MIMAT0028117
hsa-miR-642a-3p	MIMAT0020924
hsa-miR-6727-5p	MIMAT0027355
hsa-miR-4763-3p	MIMAT0019913
hsa-miR-6791-5p	MIMAT0027482
hsa-miR-5001-5p	MIMAT0021021
hsa-miR-4270	MIMAT0016900
hsa-miR-1290	MIMAT0005880
hsa-miR-371b-5p	MIMAT0019892
hsa-miR-1229-5p	MIMAT0022942
hsa-miR-3663-3p	MIMAT0018085
hsa-miR-4787-5p	MIMAT0019956
hsa-miR-6752-5p	MIMAT0027404
hsa-miR-1275	MIMAT0005929
hsa-miR-4534	MIMAT0019073
hsa-miR-7108-5p	MIMAT0028113
hsa-miR-6722-3p	MIMAT0025854
hsa-miR-2392	MIMAT0019043
hsa-miR-3940-5p	MIMAT0019229
hsa-miR-4634	MIMAT0019691
hsa-miR-1227-5p	MIMAT0022941
hsa-miR-718	MIMAT0012735
hsa-miR-4433b-3p	MIMAT0030414
hsa-miR-5585-3p	MIMAT0022286
hsa-miR-6789-5p	MIMAT0027478
hsa-miR-1471	MIMAT0007349
hsa-miR-3667-5p	MIMAT0018089
hsa-miR-4739	MIMAT0019868
hsa-miR-6741-5p	MIMAT0027383
hsa-miR-4532	MIMAT0019071
hsa-miR-765	MIMAT0003945
hsa-miR-6833-5p	MIMAT0027566
hsa-miR-4430	MIMAT0018945
hsa-miR-6751-3p	MIMAT0027403
hsa-miR-135a-3p	MIMAT0004595
hsa-miR-4695-5p	MIMAT0019788
hsa-miR-2276-3p	MIMAT0011775
hsa-miR-1181	MIMAT0005826
hsa-miR-193a-5p	MIMAT0004614
hsa-miR-3137	MIMAT0015005
hsa-miR-7846-3p	MIMAT0030421
hsa-miR-4429	MIMAT0018944
hsa-miR-1914-3p	MIMAT0007890
hsa-miR-874-3p	MIMAT0004911
hsa-miR-4707-5p	MIMAT0019807

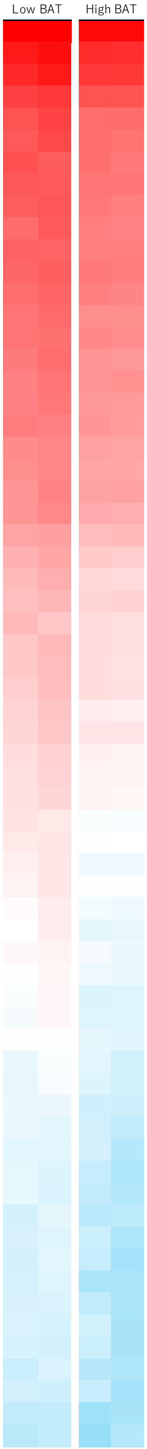

Low BAT < High BAT

mirR name	miRBase Accession No
hsa-miR-16-5p	MIMAT0000069
hsa-let-7f-5p	MIMAT0000067
hsa-miR-133b	MIMAT0000770
hsa-let-7i-5p	MIMAT0000415
hsa-let-7g-5p	MIMAT0000414
hsa-let-7a-5p	MIMAT0000062
hsa-let-7c-5p	MIMAT0000064
hsa-let-7b-5p	MIMAT0000063
hsa-miR-15b-5p	MIMAT0000417
hsa-miR-26b-5p	MIMAT0000083
hsa-miR-125b-5p	MIMAT0000423
hsa-miR-30b-5p	MIMAT0000420
hsa-let-7d-5p	MIMAT0000065
hsa-miR-378i	MIMAT0019074
hsa-miR-150-5p	MIMAT0000451
hsa-miR-195-5p	MIMAT0000461
hsa-miR-206	MIMAT0000462
hsa-miR-145-5p	MIMAT0000437
hsa-miR-23b-3p	MIMAT0000418
hsa-miR-30c-5p	MIMAT0000244
hsa-miR-133a-3p	MIMAT0000427
hsa-miR-378a-3p	MIMAT0000732
hsa-miR-99a-5p	MIMAT0000097
hsa-miR-10b-5p	MIMAT0000254
hsa-miR-342-3p	MIMAT0000753
hsa-let-7e-5p	MIMAT0000066
hsa-miR-365a-3p	MIMAT0000710
hsa-miR-200b-3p	MIMAT0000318
hsa-miR-100-5p	MIMAT0000098
hsa-miR-152-3p	MIMAT0000438
hsa-miR-10a-5p	MIMAT0000253
hsa-miR-125a-5p	MIMAT0000443
hsa-miR-378d	MIMAT0018926
hsa-miR-146b-5p	MIMAT0002809
hsa-miR-494-3p	MIMAT0002816
hsa-miR-4485-5p	MIMAT0032116
hsa-miR-96-5p	MIMAT0000095
hsa-miR-205-5p	MIMAT0000266
hsa-miR-30e-3p	MIMAT0000693
hsa-miR-203a-3p	MIMAT0000264
hsa-miR-133a-5p	MIMAT0026478
hsa-miR-335-5p	MIMAT0000765
hsa-miR-378a-5p	MIMAT0000731
hsa-miR-4299	MIMAT0016851
hsa-miR-214-3p	MIMAT0000271
hsa-miR-378g	MIMAT0018937
hsa-miR-200c-3p	MIMAT0000617
hsa-miR-28-5p	MIMAT0000085
hsa-miR-1469	MIMAT0007347
hsa-miR-204-5p	MIMAT0000265
hsa-miR-8485	MIMAT0033692
hsa-miR-194-5p	MIMAT0000460
hsa-miR-326	MIMAT0000756

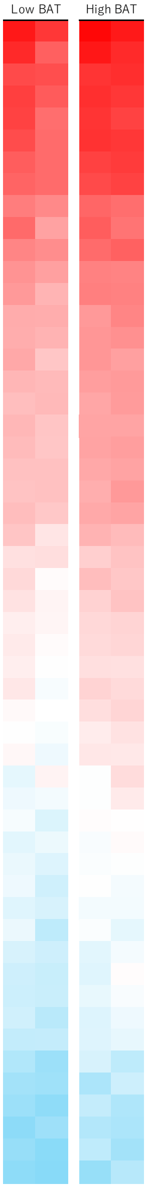

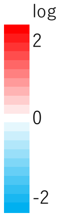

Supplement: Supplementary file 1 — Supplementary Figure S1 [file 41598_2019_49754_MOESM1_ESM.pdf]
